# Skin Transplantation

**Published:** 1898-02-26

**Authors:** 


### SKIN TRANSPLANTATION.

Dr. Taylor, of Nottingham, relates an interesting case in which, having excised a portion of skin from the upper eyelid for the relief of ptosis he found he had done too much, and finding it impracticable to get a covering for the gap by transplanting a piece of skin with a pedicle, he merely replaced the skin which he had taken away. The skin "took kindly to its old quarters." Several other cases are mentioned in which skin has been successfully transplanted after being entirely severed from all connexion with the body. "The points to be attended to in all such operations are to clear away any subcutaneous tissue, insure close approximation of the edges, apply pressure, and keep the flap warm."

